# Polyketide genes in the marine sponge *Plakortis simplex*: a new group of mono-modular type I polyketide synthases from sponge symbionts

**DOI:** 10.1111/1758-2229.12081

**Published:** 2013-07-25

**Authors:** Gerardo Della Sala, Thomas Hochmuth, Valeria Costantino, Roberta Teta, William Gerwick, Lena Gerwick, Jörn Piel, Alfonso Mangoni

**Affiliations:** 1 Dipartimento di Farmacia, Università di Napoli Federico IIVia Domenico Montesano, 49, 80131, Napoli, Italy; 2 Institute of Microbiology, Eidgenoessische Technische Hochschule (ETH) ZurichWolfgang-Pauli-Str. 10, 8093, Zurich, Switzerland; 3 Center for Marine Biotechnology and Biomedicine Scripps Institution of Oceanography Skaggs School of Pharmacy and Pharmaceutical Sciences, University of California at San Diego9500 Gilman Drive, MC 0212, La Jolla, CA, 92093-0212, USA

## Abstract

**Summary:**

**Sponge symbionts are a largely unexplored source of new and unusual metabolic pathways. Insights into the distribution and function of metabolic genes of sponge symbionts are crucial to dissect and exploit their biotechnological potential. Screening of the metagenome of the marine sponge *Plakortis simplex* led to the discovery of the *swf* family, a new group of mono-modular type I polyketide synthase/fatty acid synthase (PKS/FAS) specifically associated with sponge symbionts. Two different examples of the swf cluster were present in the metagenome of *P. simplex*. A third example of the cluster is present in the previously sequenced genome of a poribacterium from the sponge *Aplysina aerophoba* but was formerly considered orthologous to the *wcb/rkp* cluster. The *swf* cluster was also found in six additional species of sponges. Therefore, the *swf* cluster represents the second group of mono-modular PKS, after the *supA* family, to be widespread in marine sponges.**

**The putative *swf* operon consists of *swfA* (type I PKS/FAS), *swfB* (reductase and sulphotransferase domains) and *swfC* (radical S-adenosylmethionine, or radical SAM). Activation of the acyl carrier protein (ACP) domain of the SwfA protein to its holo-form by co-expression with Svp is the first functional proof of *swf* type genes in marine sponges. However, the precise biosynthetic role of the *swf* clusters remains unknown.**

## Introduction

Polyketides are the most important class of small-molecule natural products. Despite originating from a few simple building blocks such as acetyl coenzyme A (CoA) and malonyl-CoA, polyketides show a bewildering structural diversity and a broad array of biological activities, including antibiotic (e.g. erythromycin), antitumour (e.g. bryostatins) and immunosuppressant (e.g. rapamycin) actions. Most non-aromatic polyketides are synthesized by modular type I polyketide synthases (PKSs), which are large multi-domain proteins acting in a non-iterative way, each module being used only once during the biosynthesis. Two basic classes of multimodular PKSs exist, *cis*-AT PKSs, in which an acyltranferase (AT) domain is included in the multi-domain core protein, and *trans*-AT PKSs, in which the AT function is encoded by a separate gene and used iteratively.

Iterative type I PKSs are also known, in which the same set of active sites is used repeatedly. They show a single modular architecture with linear organization of active site domains similar to that of mammalian fatty acid synthases (FASs). For these enzymes, the auxiliary domains [ketoreductase (KR), dehydratase (DH), enoylreductase (ER) and others] are not involved in every condensation cycle but participate selectively in specific cycles in a predetermined manner, to yield a wide array of possible structures (Hertweck, 2009). Iterative type I PKSs are mainly found in fungi, where they are responsible of the biosynthesis of important compounds such as lovastatin (Campbell and Vederas, 2010). Other than in fungi, iterative type I PKS have been sporadically found in bacteria (Jenke-Kodama *et al*., 2005). Among animals, iterative type I PKSs have been discovered in sea urchins (Calestani *et al*., 2003), while putative non-FAS iterative type I PKS with unknown functions are present in the genomes of chickens and fish (Castoe *et al*., 2007) and in the lancelet *Branchiostoma floridae* (XP_002610053).

Marine sponges are a prolific source of polyketides. There is strong evidence that several polyketides isolated from marine sponges, such as onnamide (Piel *et al*., 2004a), psymberin (Fisch *et al*., 2009) and swinholide A (Bewley *et al*., 1996; Andrianasolo *et al*., 2005), as well as the branched fatty acids that are widely present in marine sponges (Gillan *et al*., 1988; Hochmuth *et al*., 2010), are produced by symbiotic microorganisms, which for some species, including *Plakortis simplex*, may comprise as much as 40% of the sponge volume (Vacelet, 1975; Laroche *et al*., 2007). Besides being abundant, sponge microbiota are highly specific and comprise many new candidate genera and even phyla of microorganisms (e.g. *Poribacteria*; Fieseler *et al*., 2004). The ecological relationship of these microorganisms with the sponge is still unclear, but it is generally acknowledged that most of them cannot survive *in vitro* (Uria and Piel, 2009), making it difficult to study and exploit their remarkable biosynthetic capabilities.

In the process of studying the biosynthesis of plakortin (an antimalarial polyketide peroxide present in large amounts in the marine sponge *P. simplex*) (Higgs and Faulkner, 1978) using a cultivation-independent approach (Uria and Piel, 2009), we screened the collective genome of the sponge and its symbionts (i.e. the metagenome) for PKSs. While the putative gene for plakortin biosynthesis could not be identified, an unexpected result was the discovery of Swf, a new group of mono-modular type I PKS/FAS, which appears to be specifically associated with sponge symbionts.

## Results and discussion

### Isolation of the clusters

For the screening of a metagenome, degenerate polymerase chain reaction (PCR) primers targeting conserved motifs of the ketosynthase (KS) domain of modular PKS (so-called type I PKS) (Hertweck, 2009) have been successfully used in the past (Piel, 2002; Piel *et al*., 2004b). However, for sponge metagenomes, the search for PKS biosynthetic genes for secondary metabolites has been severely hampered by a group of type I PKS that are present at very high quantities and diversity. These genes – named *sup* for *s*ponge symbiont *u*biquitous *p*ks *–* dominate sponge metagenomic DNA libraries to a such extent that PCR screening using degenerate KS primers almost invariably leads to the isolation of clones containing a *sup* cluster (Fieseler *et al*., 2007; Hochmuth and Piel, 2009).

Therefore, we used a different approach, and designed degenerate primers AT1F and AT3R2, targeting the conserved regions FPGQGsQW and QGEIAAA, respectively, of the AT module of a type I PKS. Using these AT primers, we amplified and analysed 12 AT domain DNA sequences (ca. 290 bp) from the metagenome of *P. simplex*. Eight of these sequences showed high similarity with *sup* genes, but the remaining four (PSAT_PCR01, PSAT_PCR14, PSAT_PCR20 and PSAT_PCR28) were not part of a *sup* cluster and appeared closely related to each other. In a protein Basic Local Alignment Search Tool (BLASTp) search, the proteins deduced from these four sequences were all highly identical (77–84% identical) to the open reading frame (ORF) POR_0547 from the genome of a sponge symbiont (Siegl *et al*., 2011) (see below), while showing a remarkably lower similarity to any other protein in the databases.

A large-insert 8000 clone fosmid library was then constructed from the metagenome of *P. simplex* and screened by PCR using the same AT primers. One positive clone (pPS11G3) was found and completely shotgun sequenced. The PKS gene cluster (PS11G3) was located on two contigs interrupted by a small gap, which was closed by primer walking. Because a small portion of the cluster was missing on the pPS11G3 insert, two clones containing the whole cluster were found by screening a larger metagenomic fosmid library of ∼ 245 000 clones, and the sequence of PS11G3 was completed by primer walking. Screening of this large library also led to the isolation of another fosmid, pPSA11D7, containing a second, different cluster (PSA11D7), which was also shotgun sequenced. The sequence of PSA11D7 was completed by primer walking after isolating further clones containing the cluster.

**Figure 1:**
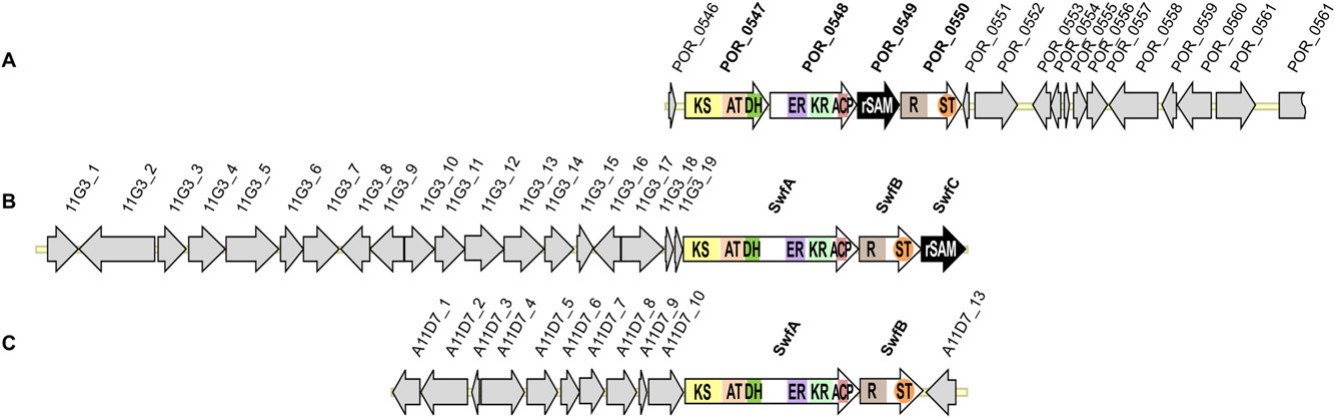
Genomic contexts and domain organization of the *swf* gene clusters.A. POR_0547–POR_0550.B. PS11G3.C. PSA11D7.

Conserved domain analysis of the PS11G3 and PSA11D7 inserts clearly showed that they encoded a type I PKS system. Recently, Hentschel and coworkers published a very similar PKS operon (ORFs POR_0547–POR_0550) isolated from a single bacterial cell of an ubiquitous sponge symbiont of the candidate phylum ‘*Poribacteria*’ (Siegl *et al*., 2011). The sequence similarities among homologous parts of the three PKS clusters are moderate to high (58–73% identity over ∼ 1000 aa, Tables 1 and 2), and also the PKS domains are the same, although the arrangement of the ORFs is different (Fig. 1).

**Table 1:** Putative genes identified on the genomic fragment pPS11G3 (*P. simplex*). Genes encoding enzymes in the ‘sponge (symbiont) widespread fatty acid synthases’ (Swf) cluster are bolded

ORF	Position (nt)	Number of aa	Putative function	Closest homologue (accession number) organism	Expect value	Identity/positives (% aa)
ORF1	515–1783	423	Aminotransferase	HNE_2588 (YP_761278), *Hyphomonas neptunium* ATCC 15444	4e-44	31/51
ORF2	4873–1796	1025	TonB-dependent receptor	Acid_6465(YP_827674), Candidatus *Solibacter usitatus* Ellin6076	0.0	43/60
ORF3	5016–6143	375	Hypothetical protein	SRM_00098 (YP_003569971), *Salinibacter ruber* M8	4e-75	41/53
ORF4	6241–7743	500	D-glutamate deacylase	Acid345_3040 (YP_592115), Candidatus *Koribacter versatilis* Ellin345	4e-140	48/63
ORF5	7792–9927	711	Peptidase S9	A3SI_03433 (YP_002762158), *Nitritalea halalkaliphila* LW7	0.0	52/71
ORF6	10017–10904	295	β-lactamase	Rhom172_1530(YP_004825287), *Rhodothermus marinus* SG0.5JP17-172	4e-81	48/62
ORF7	10922–12394	490	β-lactamase	SinacDRAFT_5672 (ZP_09567976), *Singulisphaera acidiphila* DSM 18658	4e-79	43/56
ORF8	13621–12410	403	β-lactamase	SinacDRAFT_1225 (EHO65342), *Singulisphaera acidiphila* DSM 18658	4e-44	31/49
ORF9	14889–13618	423	Hypothetical protein	HMPREF0765_1491 (ZP_03967296), *Sphingobacterium spiritivorum* ATCC 33300	5e-71	35/55
ORF10	15102–16220	372	FAD-dependent oxidoreductase	Acid_5446 (YP_826678.1), Candidatus *Solibacter usitatus*Ellin6076	7e-99	51/62
ORF11	16245–17453	402	Aminomethyltransferase	Acid345_1269 (YP_590345), Candidatus *Koribacter versatilis* Ellin345	2e-122	49/65
ORF12	17453–19066	537	FAD-dependent oxidoreductase	Acid345_1270 (YP_590346), Candidatus *Koribacter versatilis*Ellin345	0.0	66/82
ORF13	19063–20667	534	FAD-dependent oxidoreductase	Acid345_1270 (YP_590346), Candidatus *Koribacter versatilis* Ellin345	2e-130	41/60
ORF14	20708–21874	388	Aminomethyltransferase	GB2207_03824 (ZP_01224676), gamma proteobacterium HTCC2207	2e-146	55/73
ORF15	21969–22625	218	Anti-anti-sigma regulatory factor	(ACY25442), uncultured microorganism from *Aplysina aerophoba* (aa 13–111)	1e-15	40/61
			Hypothetical protein	POR_0546 (ZP_06385950) Candidatus *Poribacteria* sp. WGA-A3 (aa 136–195)	1e-15	44/72
ORF16	23738–22662	358	Alcohol dehydrogenase	HMPREF0017_00813 (ZP_06068892), *Acinetobacter lwoffii* SH145	2e-131	51/71
ORF17	23842–25515	557	Permease	SupE (ABE03914), *Aplysina aerophoba* bacterial symbiont clone pAPKS18	2e-136	44/64
ORF18	25597–25944	115	Anti-anti-sigma regulatory factor	(ACY25442), uncultured microorganism from *Aplysina aerophoba*	3e-13	35/58
ORF19	25987–26265	92	Hypothetical protein	POR_0546 (ZP_06385950), Candidatus *Poribacteria* sp. WGA-A3	8e-21	48/72
**SwfA**	**26422–33471**	**2349**	**Type I PKS (KS AT DH ER KR ACP)**	**POR_0547 (ZP_06385951), Candidatus *Poribacteria* sp. WGA-A3 (aa 1–1108)**	**0.0**	**70/83**
				**POR_0548 (ZP_06385952), Candidatus *Poribacteria* sp. WGA-A3 (aa 1105–2326)**	**0.0**	**63/76**
**SwfB**	**33468–35987**	**839**	**R** **+** **ST domains**	**POR_0550 (ZP_06385954), Candidatus *Poribacteria* sp. WGA-A3**	**0.0**	**59/74**
**SwfC**	**36003–37760**	**585**	**Radical SAM**	**POR_0549 (ZP_06385953), Candidatus *Poribacteria* sp. WGA-A3**	**0.0**	**65/79**

ACP, acyl carrier protein; AT, acyltransferase; DH, dehydratase; ER, enoylreductase; FAD, flavin adenine dinucleotide; KR, ketoreductase; KS, ketosynthase; ORF, open reading frame; PKS, polyketide synthase; R, reductase; SAM, S-adenosylmethionine; ST, sulphotransferase.

**Table 2:** Putative genes identified on the genomic fragment pPSA11D7 (*P. simplex*). Genes encoding enzymes in the ‘sponge (symbiont) widespread fatty acid synthases’ (Swf) cluster are bolded

ORF	Position (nt)	Number of aa	Putative function	Closest homologue (accessionnumber) organism	Expect value	Identity/positives (% aa)
ORF1	1564–488	358	Glycosyl transferase	ZOD2009_15156 (ZP_08045396), *Haladaptatus paucihalophilus* DX253	5e-63	36/53
ORF2	3534–1570	654	Hypothetical protein	Y11_36911 (CBY28839), *Yersinia enterocolitica* subsp. *palearctica* Y11	3e-78	31/49
ORF3	3985–3695	96	Hypothetical protein	sce5011 (YP_001615654), *Sorangium cellulosum* ‘So ce 56’	2e-17	50/69
ORF4	4123–5823	566	HNH endonuclease	Anae109_1700 (YP_001378888), *Anaeromyxobacter* sp. Fw109-5	1e-14	54/71
ORF5	5926–7167	413	Integrase/recombinase	ROS217_01170 (ZP_01038402.1), *Roseovarius* sp. 217	2e-114	49/66
ORF6	7161–8072	303	Integrase/recombinase	XerC (P_002540039), *Agrobacterium vitis* S4	3e-103	55/70
ORF7	8069–9061	330	Integrase/recombinase	KKY_3614 (YP_004901348), *Pelagibacterium halotolerans* B2	6e-171	72/86
ORF8	10431–9385	348	NAD-dependent epimerase/dehydratase	Hoch_1681(YP_003266124), *Haliangium ochraceum* DSM	8e-36	35/48
ORF9	10469–10784	104	Hypothetical protein	SeloA3_010100011807(ZP_09956060) *Sphingomonas elodea* ATCC 31461	7e-11	36/60
ORF10	10837–12291	484	Sugar transporter	ED21_29266(ZP_0186498), *Erythrobacter* sp. SD-21	3e-79	37/53
**SwfA**	**12302–19387**	**2361**	**Type I PKS (KS AT DH ER KR ACP)**	**POR_0547 (ZP_06385951), Candidatus *Poribacteria* sp. WGA-A3 (aa 3–1111)**	**0.0**	**73/84**
				**POR_0548 (ZP_06385952), Candidatus *Poribacteria* sp. WGA-A3 (aa 1118–2345)**	**0.0**	**65/77**
**SwfB**	**19396–21894**	**832**	**R** **+** **ST domains**	**POR_0550 (ZP_06385954), Candidatus *Poribacteria* sp. WGA-A3**	**0.0**	**58/73**
ORF13	22917–21928	329	Oxidoreductase	GobsU_010100005149 (ZP_02731160), *Gemmata obscuriglobus* UQM 2246	1e-49	35/52

ACP, acyl carrier protein; AT, acyltransferase; DH, dehydratase; ER, enoylreductase; KR, ketoreductase; KS, ketosynthase; NAD, nicotinamide adenine dinucleotide; ORF, open reading frame; PKS, polyketide synthase; R, reductase; ST, sulphotransferase.

In the absence of any close homologue, POR_0547 and POR_0548 had been designated in the original study as belonging to a WcbR-type PKS. The *wcb* clusters (and their orthologous *rkp* clusters) (Kiss *et al*., 1997; Parada *et al*., 2006; Donadio *et al*., 2007) are involved in lipopolysaccharide biosynthesis and are found in α-Proteobacteria, which can induce root nodule formation in plants (for instance bacteria of the taxon Rhizobiales). Additionally, other bacteria living in symbiosis with plants contain these genes, especially β-Proteobacteria: the clusters in *Burkholderia* are designated as *wcb*, while those in *Nitrosomonas* (and *Rhizobium*) are called *rkp*, most of the genes of *wcb* and *rkp* clusters being homologues. Beside the type I PKS gene (*wcbR*/*rkpA*), the cluster comprises several genes for the biosynthesis and the export of capsular polysaccharides.

However, the discovery of PS11G3 and PSA11D7 shows that the assignment of POR_0547 and POR_0548 to a WcbR-type PKS needs to be revised. The similarity to WcbR is only moderate (POR_0547: 96% coverage, 33% identity with NP_841435; POR_0548: 65% coverage, 35% identity with EGD01311). In addition, comparable similarities are observed to very different type I PKS enzymes, such as those involved in the secondary metabolism (e.g. 32% identity of POR_0548 with AnaF from the anatoxin-a synthetase gene cluster). These data, together with the close homology between the three gene clusters, the analogy of their domains and the absence of the other genes normally present in the *wcb* clusters, suggest that the clusters belong to a separate group of PKSs, with no particular relationship to the *wcb* cluster. We propose the name *swf* (‘sponge (symbiont) widespread FASs’), in contrast to the *sup* genes (Fieseler *et al*., 2007), the other group of type I PKS/FAS that are widespread and abundant in sponge metagenomes.

### In silico analysis of the swf gene clusters

#### Organization and genomic contexts

The putative *swf* PKS/FAS operon is composed of three genes, *swfA*, *swfB* and *swfC* (not present in PSA11D7) (Fig. 1).

The PKS gene (*swfA*) contained in the library fosmid pPS11G3 has a very high similarity to a fusion of POR_0547 and POR_0548. It is followed by *swfB*, which encodes for thioester reductase (R) and sulphotransferase (ST) domains (high homology to POR_0550), and by *swfC*, which encodes for a putative radical SAM enzyme (high homology to POR_0549). The two latter ORFs have changed their relative positions compared with the POR operon. The *swf* cluster found in the library fosmid pPSA11D7 contains only *swfA* and *swfB* (each highly similar to their respective homologues in PS11G3 and POR), while a gene for a radical SAM, which would be homologous to *swfC* (PS11G3) and POR_0549, is not present.

BLASTp searches (Tables 1 and 2) revealed that, in addition to these shared features, no similarities were present among PS11G3, PSA11D7 and POR, nor similarities to *wcb*/*rkp*.

#### SwfA

The SwfA proteins are each composed of only one complete PKS module, indicative of either an iterative mode of action or a single elongation. The domain organization of SwfA is KS-AT-DH-ER-KR-ACP. From the domain organization of SwfA, a saturated acyl chain product is expected, although a (poly)unsaturated and/or (poly)hydroxylated acyl chain cannot be excluded, because in some iterative PKSs the reduction domains may be optionally used during each of the elongation steps (Hertweck, 2009).

**Figure 2:**
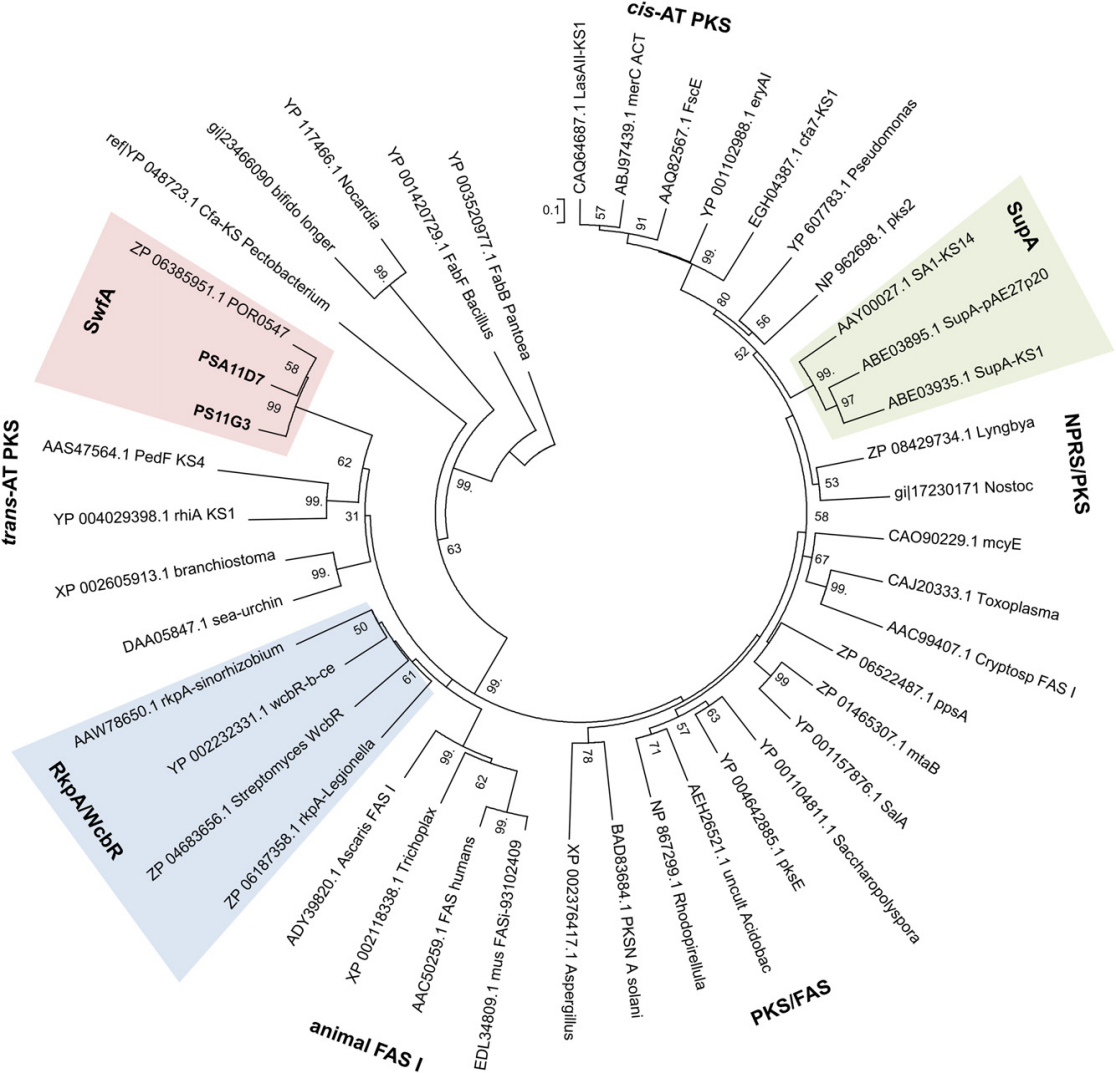
Neighbour-joining tree of full length KS domains from type I FAS, *cis*-AT PKS, *trans*-AT PKS, Sup (sponge symbiont ubiquitous PKS), PKS/FAS, PKS/NRPS, RkpA/WcbR. The KS tree is rooted with the type II KS FabB from *P. annatis* LMG20103. Bootstrap values are given at the nodes.

SwfA starts with a KS domain (∼ 420 aa) with moderate homology to type I PKSs from various organisms. Phylogenetic analysis (KS tree rooted with the type II KS FabB from *Pantoea annatis* LMG20103) shows a clear separation of the new sponge-derived SwfA enzymes from the WcbR/RkpA sequences (Fig. 2; see also Fig. S1). The KS domain of SwfA shows features that are not shared by any other KS domains, including WcbR/RkpA (Fig. S2). In addition, the second ‘Q’ in the DPQQR motif is replaced by either ‘I’ or ‘V’, which is similar to animal FAS I and not to PKSs (all *cis*-AT and WcbR/RkpA sequences possess an intact DPQQR motif), and the motif HGTGT of *cis*-AT PKS and WcbR/RkpA sequences is changed to HATGT. This explains why *swf* sequences may be underrepresented or absent in previous PCR screenings for PKSs in sponge metagenomes (Schirmer *et al*., 2005; Fieseler *et al*., 2007), which targeted the standard DPQQR and HGTGT motifs.

For the AT domain, all SwfA homologues share the motif IAFH, which suggests malonyl-CoA as the substrate (Fig. S3) (Reeves *et al*., 2001), but the other substrate determining motif GHS**S**GE is unusual because an amino acid larger than Ser is normally present in malonyl-CoA specific AT domains (Smith and Tsai, 2007). The QCALVEL motif (aa number 591–597) is unique to SwfA_AT_ and is also present (with some variation in the first and last aa) in the four *swf* AT fragments amplified from the metagenome of *P. simplex* with the AT1F/AT3R2 primers (see above). It appears to be a well-conserved signature motif for SwfA genes and was used to design primers to screen for the presence of the *swf* cluster in the metagenomes of other species of sponges (see below).

No useful homology information could be obtained for the DH, ER, KR and ACP domains. The BLASTp hits were heterogeneous and displayed only moderate to low homology.

#### SwfB

The ORF encodes a predicted fusion of an N-terminal thioester R domain and a C-terminal ST domain (Fig. 1). SwfB shows high similarity to the C-terminal two domains of two multimodular type I FAS of protists of the class Coccidia (Table 1), the only known homologues in which the R and ST domains are contiguous as in SwfB. Because no functional studies have been conducted with this gene, the function of SwfB cannot be inferred.

R domains are frequently present in modular enzymes of fungi, myxobacteria and cyanobacteria (Zhu *et al*., 2010). They can reductively release the assembled chain as an aldehyde (often further elaborated to primary alcohol or amine) (Du and Lou, 2010), or they can be redox-inactive catalysts of carbon chemistry, for instance to form heterocycles (Liu and Walsh, 2009). ST domains catalyse transfer of a sulphonate group to a hydroxy or amino group (Chapman *et al*., 2004). The resulting sulphate group is usually found in the final metabolite, but sulphonation may also be a means to generate a good leaving group as reported for curacin A biosynthesis (Gu *et al*., 2009), in which sulphonation of a β-hydroxy-acyl-ACP is followed by thioesterase-mediated hydrolysis, decarboxylation and sulphate elimination to give a terminal alkene.

For SwfB, the combination of R and ST modules could suggest conversion of the terminal carboxylate to a primary sulphate group.

#### SwfC

**Figure 3:**
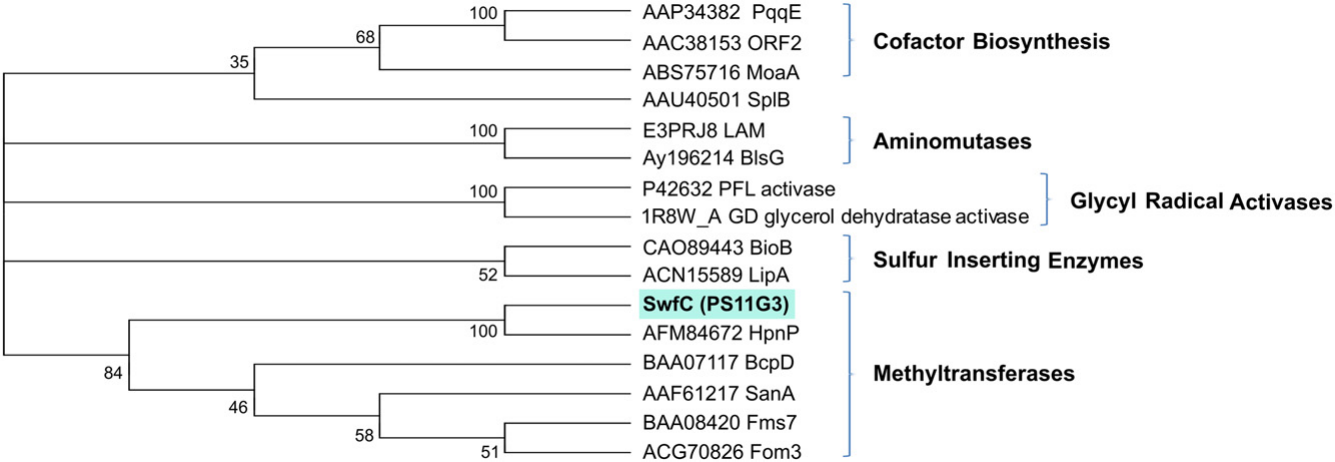
Neighbour-joining tree (condensed tree, cut-off value is 35%) displaying topology of radical SAM enzymes with known biological function. SwfC (PS11G3) clusters with radical SAM methyltransferases. Bootstrap values are given at the nodes.

SwfC shows high similarity to a number of radical SAM enzymes. It has a predicted N-terminal vitamin B12 binding domain and a central radical SAM superfamily domain, including the characteristic CX_3_CX_2_C motif. Radical SAM enzymes can catalyse an array of different reactions (Wang and Frey, 2007; Roach, 2011). The first event is always the extraction of a hydrogen radical from an unreactive C–H bond, but the final outcome of the reaction can vary greatly, including isomerizations, complex rearrangements, oxidations and methylations. In a phylogenetic tree constructed using radical SAM proteins that have been functionally characterized, SwfC clusters with proteins that act as methyltransferases (Fig. 3). Therefore, SwfC might act as a methyltransferase as well. The closest homologue of SwfC in the tree is an enzyme (HpnP) responsible for 2-methylation of hopanoids (Welander *et al*., 2010). While a similar enzymatic activity must be present in some of the bacterial symbionts of *P. simplex*, because 12-methylhopanoids have been found in large amounts in the extract of this sponge (Costantino *et al*., 2000), it appears unlikely that SwfC is involved in this specific activity.

### Widespread distribution of the swf cluster

For a preliminary analysis of the distribution and diversity of the *swf* cluster, we designed the primers SWF_ATF and SWF_ATR from the conserved regions FSGQGTQW and QCALVEL, respectively, of the AT domain of SwfA proteins. Using these primers, we amplified by PCR this fragment from the metagenome of *P. simplex* as well as from six ‘high microbial abundance sponges’ (Hentschel *et al*., 2006), namely *Aplysina fulva*, *Aiolochroia crassa*, *Smenospongia aurea*, *Xestospongia muta*, *Ircinia felix* and *Theonella swinhoei*. In all cases, the PCR product showed the expected length (about 220 bp) for a *swfA* fragment (Fig. S6). The PCR products were subcloned, and six clones from each species were sequenced. All the deduced protein fragments showed a very high similarity to SwfA; each amplicon showed 64–97% identity to each of the three SwfA homologues described in this paper. In addition, a BLASTp search found POR_0547 as the first hit for all the sequences (E value < 10^−26^), while all the other hits were heterogeneous and showed a much lower similarity (at most 51% identity, E-value > 10^−13^).

These data clearly show that all the amplicons were from *swfA* genes and therefore, that the *swf* cluster was present in all the sponges studied. Therefore, the *swf* cluster appears to be widespread in marine sponges.

### Functional study of the Swf enzymes

#### *In vivo* phosphopantetheinylation of SwfA_ACP_

**Figure 4:**
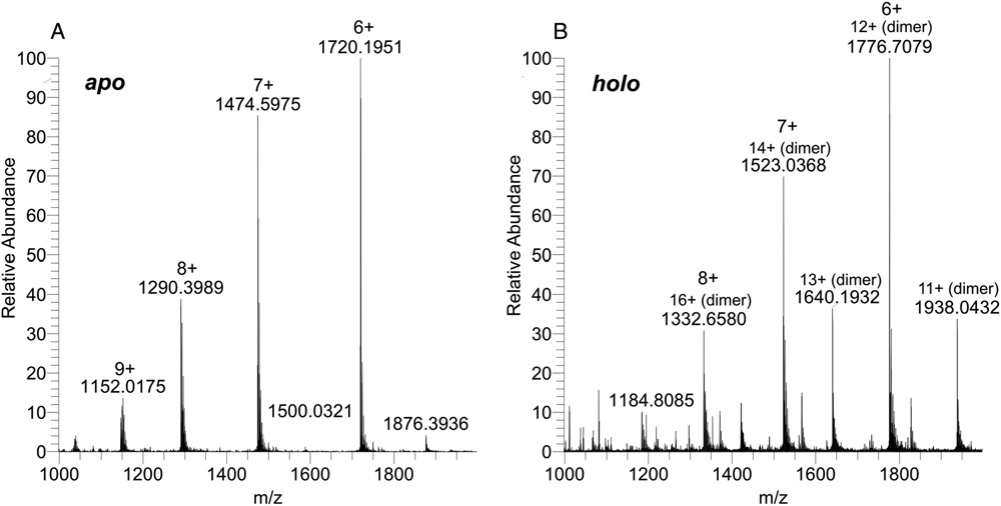
A. High-resolution ESI-MS spectrum of *apo*-SwfA_ACP_.B. High-resolution ESI-MS spectrum of *holo*-SwfA_ACP_. The peaks of the *holo*-protein are overlapped with those of its dimer (see also Fig. S5). Odd-charged peaks of the dimer are also present. The 339 amu difference between corresponding peaks of *apo*- and *holo*-SwfA_ACP_ accounts for a phosphopantetheine adduct (but see text).

The ACP domain of SwfA (SwfA_ACP_) was expressed with and without co-expression of a gene for the 4′-phosphopantetheinyl transferase (PPTase) Svp from the bleomycin-producing *Streptomyces verticillus* ATCC15003 (Sanchez *et al*., 2001). The high-resolution electrospray ionization (ESI) mass spectra of the two purified enzyme preparations were compared to test whether the *apo*-SwfA_ACP_ could be activated to its *holo*-form *in vivo*. For each of the two expressed proteins, several pseudomolecular ion peaks with different charges were observed, the 6 + charge state being the most abundant (Fig. 4). These peaks were clearly resolved to isotopic peaks (Fig. S5), and the most intense isotopic peaks for each ion were measured at *m*/*z* 1720.1949 and 1776.7079, respectively, for the *apo*- and *holo*-form. The measured *m*/*z* values were in excellent agreement with the respective theoretical values (calculated using IsoPro 3.0, MS/MS software; Senko, 2003) at *m*/*z* 1720.1972 (*apo*-form, C_449_H_704_N_134_O_140_S_3_, error 1.2 ppm, monoisotopic mass of the uncharged protein 10309.1230 amu) and 1776.7101 (*holo*-form, C_460_H_724_N_136_O_146_PS_4_, 1.2 ppm, 10648.2021 amu). The mass of the *holo*-form is higher by 339.0791, which is the expected change for a phosphopantetheine adduct.

It should be noted that, while the experimentally well-established mass change for phosphopantetheinylation is 339 (Byers and Ward, 2003; Sigma-Aldrich, 2012), the expected mass change should be 340 (358 for phosphopantetheine in the neutral dihydrogenophosphate form minus 18 for the loss of a water molecule). A mass change by 339 corresponds to an additional C_11_H_20_N_2_O_6_PS neutral fragment, which for the nitrogen rule must be an odd-electron fragment and therefore implies a radical. To the best of our knowledge, this discrepancy has never been previously noticed nor explained. A possible explanation is that the terminal SH of the phosphopantetheine group may be oxidized, yielding a disulphide dimer (this dimer was indeed observed in the ESI mass spectrum of *holo*-SwfA_ACP_, Fig. S5). In the ESI source, the usual (poly)protonation reactions may be accompanied by homolytic cleavage of the disulphide bond, yielding two sulphur radicals.

The comparison of the mass spectra of SwfA_ACP_ with and without Svp co-expression showed that in the absence of Svp, only the *apo*-form of the ACP was present. In contrast, the co-expression with Svp resulted in a complete conversion of the *apo*- into the *holo*-form. This is the first functional information about the *swf* cluster, demonstrating that the predicted ACP domain folds correctly, is recognized by PPTase, and is therefore presumably functional. In addition, these results demonstrate that ACPS (the PPTase of the *E. coli* expression host) is unable to catalyse phosphopantetheinylation, and therefore, that in functional studies of *swf* in *E. coli*, co-expression of the Svp PPTase is essential.

#### Heterologous expression and liquid chromatography-mass spectrometry (LC-MS) analysis

For heterologous expression of the *swf* cluster, the whole cluster (PS11G3 type) was cloned in the expression vector pHIS8-Svp (Jez *et al*., 2000; Izumikawa *et al*., 2006), yielding the recombinant plasmid pGS38 (see Supporting Information for details). After cloning, the new recombinant plasmid was transferred into *E. coli* BAP1 (Pfeifer *et al*., 2001) by electroporation in order to perform heterologous expression under control of the T7 promoter.

The extracts of the transformants containing the *swf* cluster and of their culture broths were analysed by liquid chromatography/high resolution electrospray ionization mass spectrometry (LC-HR-ESI-MS) and compared with the extracts from negative controls (i.e. clones grown in presence of the inducer, but containing only the expression vector pHIS8-Svp without the *swf* insert). All the experiments were performed in triplicate, and both positive- and negative-ion mass spectra were recorded. Although differences were observed in the relative amounts of some metabolites, the LC-MS data revealed no compounds that were present in all the transformants and absent in all the negative controls.

### Implications and outlook

Sponge symbionts are a largely unexplored source of new and unusual metabolic pathways. Insights into the distribution and function of metabolic genes of sponge symbionts are crucial to dissect and exploit their biotechnological potential, which could pave the way to the sustainable production of molecules of practical interest.

The *swf* cluster represents the second example, after the *sup* cluster, of a PKS/FAS cluster that is to date only found in bacterial symbionts of sponges. The *swf* cluster has been found in *P. simplex* and *Aplysina aerophoba* symbionts. In addition, PCR amplification of metagenomic DNA from six additional and taxonomically distant species of sponge with primers designed for *swf* produced amplicons that showed high sequence similarities to the AT domain of *swfA*. Therefore, the *swf* cluster appears to be widespread in marine sponges.

In *A. aerophoba*, *swf* has been shown to be hosted by poribacteria (Siegl *et al*., 2011), and the same may hold true for *P. simplex* (which is known to contain poribacteria) and for the other species studied, although no positive evidence about this exists at present. Poribacteria are considered a possible ancient bacterial symbiont of marine sponges, but little is known about them because they cannot be cultivated. The single amplified genome derived from a member of the *Poribacteria* was sequenced and analysed by sequence homology to gain information on the lifestyle of these sponge-specific bacteria (Siegl *et al*., 2011). Because POR_0547 and POR_0548 appeared related with *wcbR*, and some *wcbR* homologues were shown to be major symbiosis and virulence factors in their producer, it was suggested that the product of these poribacterial genes could exert a similar role in the sponge–microbe interaction (Siegl *et al*., 2011). Now, however, it is clear that the genes (along with the two downstream ORFs) actually belong to *swf*, suggesting a different function.

The putative *swf* operon consists of *swfA* (type I PKS/FAS), *swfB* (R and ST domains) and *swfC* (radical SAM). SwfA is a new type of mono-modular type I PKS/FAS that has only modest homology to WcbR or RkpA. The deduced product of the single PKS module (KS-AT-DH-ER-KR-ACP), acting iteratively, is an acyl chain, either completely reduced or variously functionalized if the reduction domains are not all used during each of the elongation steps. SwfA appears split in two ORFs (POR_0547 and POR_0548) in the *A. aerophoba* symbiotic poribacterium. However, if one considers that all the other homologous genes are encoded on a single ORF (Fig. S1), and that POR_0547 and POR_0548 are not in frame, it is conceivable that a sequencing error may be the reason for this discrepancy and that also the poribacterial SwfA is actually encoded on a single ORF.

The other two proteins produced by the cluster, SwfB (thioester R and ST domains) and SwfC (radical SAM), are expected to modify the acyl chain produced by SwfA in unknown ways. While the expected product of elaboration of an acyl chain by SwfB would possibly be an alkyl sulphate that might be methylated by SwfC, no such metabolites are known to be widespread in sponges. However, alkyl sulphates would not be observed (if present) by standard analytical procedures because an alkyl sulphate would not be affected by the reactions of saponification and methylation commonly used for lipid analysis.

The actual biosynthetic function of the swf cluster remains to be elucidated. It appears unlikely that the cluster is not functional, considering that it is highly conserved (> 58% identity, 75% positives between the three examples of the swf cluster) in the symbiotic microbiota of two taxonomically distant genera of sponges such as *Plakortis* and *Aplysina*. The activation of the SwfA_ACP_ to its *holo*-form by co-expression with Svp, which is the first functional examination of the *swf* type genes in marine sponges, also supports this idea and additionally suggests that expression of a functional *swf* pathway in a heterologous host is possible. However, a first attempt of the heterologous expression of the entire *swf* cluster in *E. coli* BAP1 did not lead to the detection of any new metabolites.

### Accession codes

The pPS11G3 and pPSA11D7 complete nucleotide sequences were deposited into GenBank respectively under the accession numbers JX946307 and JX946308. The AT gene partial sequences from PCR screening were deposited into GenBank under the accession numbers KC424641 through KC424644 (primers AT1F/AT3R2), and JX946309 through JX946332 and KF241758 through KF241775 (primers SWF_ATF/SWF_ATR).

## Supporting information

Detailed experimental procedures **Fig. S1.** Unweighted Pair Group Method with Arithmetic Mean (UPGMA) tree showing phylogenetic relationships of KS-AT domains from different types of PKS and FAS enzymes. **Fig. S2.** Alignment of KS domains from type I PKSs and FASs. **Fig. S3.** Alignment of AT domains from type I PKSs and FASs. **Fig. S4.** Sodium dodecyl sulfate polyacrylamide gel electrophoresis (SDS-PAGE) gel showing the successful expression of the N-terminal His8-tag fusion proteins *apo*-SwfA_ACP_ and *holo*-SwfA_ACP_ before and after purification using nickelnitriloacetic acid (Ni-NTA) columns. **Fig. S5.** Analysis of the fine structure of the sixfold charged ion peak of *holo*-SwfA_ACP_. **Fig. S6.** PCR detection of *swf* genes in seven different ‘high microbial abundance’ marine sponges.

## References

[b1] Andrianasolo EH, Gross H, Goeger D, Musafija-Girt M, McPhail K, Leal RM (2005). Isolation of swinholide A and related glycosylated derivatives from two field collections of marine cyanobacteria. Org Lett.

[b2] Bewley CA, Holland ND, Faulkner DJ (1996). Two classes of metabolites from *Theonella swinhoei* are localized in distinct populations of bacterial symbionts. Cell Mol Life Sci.

[b3] Byers HL, Ward MA, Van, EJE, Dunn MJ (2003). Mass spectrometry. A powerful analytical tool. Proteomic and Genomic Analysis of Cardiovascular Disease.

[b4] Calestani C, Rast JP, Davidson EH (2003). Isolation of pigment cell specific genes in the sea urchin embryo by differential macroarray screening. Development.

[b5] Campbell CD, Vederas JC (2010). Biosynthesis of lovastatin and related metabolites formed by fungal iterative PKS enzymes. Biopolymers.

[b6] Castoe TA, Stephens T, Noonan BP, Calestani C (2007). A novel group of type I polyketide synthases (PKS) in animals and the complex phylogenomics of PKSs. Gene.

[b7] Chapman E, Best MD, Hanson SR, Wong CH (2004). Sulfotransferases: structure, mechanism, biological activity, inhibition, and synthetic utility. Angew Chem Int Ed.

[b8] Costantino V, Fattorusso E, Imperatore C, Mangoni A (2000). The first 12-methylhopanoid: 12-methylbacteriohopanetetrol from the marine sponge *Plakortis simplex*. Tetrahedron.

[b9] Donadio S, Monciardini P, Sosio M (2007). Polyketide synthases and nonribosomal peptide synthetases: the emerging view from bacterial genomics. Nat Prod Rep.

[b10] Du L, Lou L (2010). PKS and NRPS release mechanisms. Nat Prod Rep.

[b11] Fieseler L, Horn M, Wagner M, Hentschel U (2004). Discovery of the novel candidate phylum ‘Poribacteria’ in marine sponges. Appl Environ Microbiol.

[b12] Fieseler L, Hentschel U, Grozdanov L, Schirmer A, Wen G, Platzer M (2007). Widespread occurrence and genomic context of unusually small polyketide synthase genes in microbial consortia associated with marine sponges. Appl Environ Microbiol.

[b13] Fisch KM, Gurgui C, Heycke N, van der Sar SA, Anderson SA, Webb VL (2009). Polyketide assembly lines of uncultivated sponge symbionts from structure-based gene targeting. Nat Chem Biol.

[b14] Gillan FT, Stoilov IL, Thompson JE, Hogg RW, Wilkinson CR, Djerassi C (1988). Fatty acids as biological markers for bacterial symbionts in sponges. Lipids.

[b15] Gu L, Wang B, Kulkarni A, Gehret JJ, Lloyd KR, Gerwick L (2009). Polyketide decarboxylative chain termination preceded by O-sulfonation in curacin A biosynthesis. J Am Chem Soc.

[b16] Hentschel U, Usher KM, Taylor MW (2006). Marine sponges as microbial fermenters. FEMS Microb Ecol.

[b17] Hertweck C (2009). The biosynthetic logic of polyketide diversity. Angew Chem Int Ed.

[b18] Higgs MD, Faulkner DJ (1978). Plakortin, an antibiotic from *Plakortis halichondrioides*. J Org Chem.

[b19] Hochmuth T, Piel J (2009). Polyketide synthases of bacterial symbionts in sponges – evolution-based applications in natural products research. Phytochemistry.

[b20] Hochmuth T, Niederkruger H, Gernert C, Siegl A, Taudien S, Platzer M (2010). Linking chemical and microbial diversity in marine sponges: possible role for poribacteria as producers of methyl-branched fatty acids. Chembiochem.

[b21] Izumikawa M, Cheng Q, Moore BS (2006). Priming type II polyketide synthases via a type II nonribosomal peptide synthetase mechanism. J Am Chem Soc.

[b22] Jenke-Kodama H, Sandmann A, Müller R, Dittmann E (2005). Evolutionary implications of bacterial polyketide synthases. Mol Biol Evol.

[b23] Jez JM, Ferrer JL, Bowman ME, Dixon RA, Noel JP (2000). Dissection of malonyl-coenzyme A decarboxylation from polyketide formation in the reaction mechanism of a plant polyketide synthase. Biochemistry.

[b24] Kiss E, Reuhs BL, Kim JS, Kereszt A, Petrovics G, Putnoky P (1997). The rkpGHI and -J genes are involved in capsular polysaccharide production by *Rhizobium meliloti*. J Bacteriol.

[b25] Laroche M, Imperatore C, Grozdanov L, Costantino V, Mangoni A, Hentschel U, Fattorusso E (2007). Cellular localisation of secondary metabolites isolated from the Caribbean sponge *Plakortis simplex*. Mar Biol.

[b26] Liu X, Walsh CT (2009). Cyclopiazonic acid biosynthesis in *Aspergillus* sp.: characterization of a reductase-like R* domain in cyclopiazonate synthetase that forms and releases cyclo-acetoacetyl-L-tryptophan. Biochemistry.

[b27] Parada M, Vinardell JM, Ollero FJ, Hidalgo A, Gutierrez R, Buendia-Claveria AM (2006). *Sinorhizobium fredii* HH103 mutants affected in capsular polysaccharide (KPS) are impaired for nodulation with soybean and *Cajanus cajan*. Mol Plant Microbe Interact.

[b28] Pfeifer BA, Admiraal SJ, Gramajo H, Cane DE, Khosla C (2001). Biosynthesis of complex polyketides in a metabolically engineered strain of *E. coli*. Science.

[b29] Piel J (2002). A polyketide synthase-peptide synthetase gene cluster from an uncultured bacterial symbiont of Paederus beetles. Proc Nat Acad Sci USA.

[b30] Piel J, Hui D, Wen G, Butzke D, Platzer M, Fusetani N, Matsunaga S (2004a). Antitumor polyketide biosynthesis by an uncultivated bacterial symbiont of the marine sponge *Theonella swinhoei*. Proc Natl Acad Sci USA.

[b31] Piel J, Hui D, Fusetani N, Matsunaga S (2004b). Targeting modular polyketide synthases with iteratively acting acyltransferases from metagenomes of uncultured bacterial consortia. Environ Microbiol.

[b32] Reeves CD, Murli S, Ashley GW, Piagentini M, Hutchinson CR, McDaniel R (2001). Alteration of the substrate specificity of a modular polyketide synthase acyltransferase domain through site-specific mutations. Biochemistry.

[b33] Roach PL (2011). Radicals from S-adenosylmethionine and their application to biosynthesis. Curr Opin Chem Biol.

[b34] Sanchez C, Du L, Edwards DJ, Toney MD, Shen B (2001). Cloning and characterization of a phosphopantetheinyl transferase from *Streptomyces verticillus* ATCC15003, the producer of the hybrid peptide-polyketide antitumor drug bleomycin. Chem Biol.

[b35] Schirmer A, Gadkari R, Reeves CD, Ibrahim F, DeLong EF, Hutchinson CR (2005). Metagenomic analysis reveals diverse polyketide synthase gene clusters in microorganisms associated with the marine sponge *Discodermia dissoluta*. Appl Environ Microbiol.

[b36] Senko MW (2003). https://sites.google.com/site/isoproms/.

[b37] Siegl A, Kamke J, Hochmuth T, Piel J, Richter M, Liang C (2011). Single-cell genomics reveals the lifestyle of Poribacteria, a candidate phylum symbiotically associated with marine sponges. ISME J.

[b38] Sigma-Aldrich (2012). http://www.sigmaaldrich.com/life-science/proteomics/post-translational-analysis/phosphorylation/mass-changes.html.

[b39] Smith S, Tsai SC (2007). The type I fatty acid and polyketide synthases: a tale of two megasynthases. Nat Prod Rep.

[b40] Uria A, Piel J (2009). Cultivation-independent approaches to investigate the chemistry of marine symbiotic bacteria. Phytochem Rev.

[b41] Vacelet J (1975). Étude en microscopie électronique de l’association entre bactéries et spongiaires du genre Verongia (Dictyoceratida). J Microsc Biol Cell.

[b42] Wang SC, Frey PA (2007). S-adenosylmethionine as an oxidant: the radical SAM superfamily. Trends Biochem Sci.

[b43] Welander PV, Coleman ML, Sessions AL, Summons RE, Newman DK (2010). Identification of a methylase required for 2-methylhopanoid production and implications for the interpretation of sedimentary hopanes. Proc Natl Acad Sci USA.

[b44] Zhu G, Shi X, Cai X (2010). The reductase domain in a Type I fatty acid synthase from the apicomplexan *Cryptosporidium parvum*: restricted substrate preference towards very long chain fatty acyl thioesters. BMC Biochem.

