# Supplementary material for: Polyketide genes in the marine sponge Plakortis simplex: a new group of mono-modular type I polyketide synthases from sponge symbionts
Source: Environ Microbiol Rep. 2013 Jul 25;5(6):809–18. doi: 10.1111/1758-2229.12081 (PMC3908369; doi:10.1111/1758-2229.12081)
Supplement: Supplementary file 1 — Detailed experimental procedures Fig. S1. Unweighted Pair Group Method with Arithmetic Mean (UPGMA) tree showing phylogenetic relationships of KS-AT domains from different types of PKS and FAS enzymes. Fig. S2. Alignment of KS domains from type I PKSs and FASs. Fig. S3. Alignment of AT domains from type I PKSs and FASs. Fig. S4. Sodium dodecyl sulfate polyacrylamide gel electrophoresis (SDS-PAGE) gel showing the successful expression of the N-terminal His8-tag fusion proteins apo-SwfAACP and holo-SwfAACP before and after purification using nickelnitriloacetic acid (Ni-NTA) columns. Fig. S5. Analysis of the fine structure of the sixfold charged ion peak of holo-SwfAACP. Fig. S6. PCR detection of swf genes in seven different ‘high microbial abundance’ marine sponges. [file emi40005-0809-sd1.pdf]

Supporting Information

Polyketide Genes in the Marine Sponge *Plakortis simplex*: a New Group of Mono-Modular Type-I Polyketide Synthases from Sponge Symbionts

Gerardo Della Sala, Thomas Hochmuth, Valeria Costantino, Roberta Teta, William Gerwick, Lena Gerwick, Jörn Piel, Alfonso Mangoni*

alfonso.mangoni@unina.it

Detailed Experimental Procedures

General

As general bacterial strain for cloning *E. coli* XL1 Blue MRF' (Stratagene) was used. Single read sequencing, including shotgun sequencing of two library fosmids (7-fold coverage) was performed on ABI 3730XL (Applied Biosystems). The protein mass spectra were obtained with LTQ Orbitrap XL (Thermo scientific) in the FTMS positive ESI full ms mode. HR full MS experiments (positive ions) were carried out in the mass ranges *m*/*z* 200–2000 at a nominal resolving power of 100,000. Actual resolution of the spectrum varies over the whole mass range and was 63,000 at *m*/*z* 1700–1800. Calibration was performed just before the analyses by using a mixture of caffeine, MRFA (L-methionyl-arginyl-phenylalanyl-alanine acetate–H_2O) and Ultramark 1621 for the mass range m/z 800–2000. The samples were infused into the source at a flow rate of 5 µL min^{-1}, and the following source settings were used: spray voltage 3.7 kV, capillary temperature 275 °C, capillary voltage 39 V, tube lens voltage 150 V, sheath gas 5 (arbitrary units), no auxiliary gas.

Sponge collection

Specimens of *Plakortis simplex, Aplysina fulva, Aiolochroia crassa,* and *Smenospongia aurea,* were collected by scuba diving at depths of 5–15 m offshore Little San Salvador Island and/or Grand Bahamas Island, Caribbean Sea, Bahamas. Individuals were cut into pieces and immediately stored in five volumes of RNAlater (Life Tecnologies) stabilization solution. The samples were kept at –20 °C until shipped to the laboratory, then the stabilization solution was removed and the samples were kept frozen at –80 °C until used. Specimens of *Xestospongia muta* and *Ircinia felix* from Bahamas, and of *Theonella swinhoei* from Solomon Islands were immediately frozen after collection, and kept at –20 °C until used.

DNA isolation and library construction

To ~40 mg of frozen sponge (in RNA later) 700 µL of lysis buffer I (200 mM Tris-Cl, 50 mM EDTA, 1.4 M NaCl, 2% CTAB, 0.5% PVP, all in milliQ®-H_2O) were added and incubated at 37 °C for 1 h in a thermomixer (1,400 rpm). After addition of 2.8 µL β-mercaptoethanol, 70 µL 10% SDS, 2 µL RNase A (100 mg/mL), and 40 µL proteinase K (10 mg/mL) the tube was incubated at 55 °C for a further hour in a thermomixer (1,400 rpm). At this time, the microcentrifuge tube was spun 4 min at 5,000 rpm. The clear middle phase was transferred to a new microcentrifuge tube containing 750 µL $CHCl_3$ and centrifuged 10 min at 15,000 rpm. After repetition of the $CHCl_3$ wash, the supernatant was transferred to a new microcentrifuge tube containing 750 µL of 70% aqueous isopropyl alcohol containing 10% (v/v) 3M NaOAc (pH 5.5) at room temperature. The precipitated DNA was spun down at top speed for 20 min, washed with ice-cold ethanol, dried and dissolved in ~60 µL elution buffer (10 mM Tris-Cl, pH 8.5). The amount of one tube was enough for PCR screening, for library construction the protocol was upscaled to ~500 mg of frozen sponge.

Metagenomic libraries of *P. simplex* were constructed using the CopyControl Fosmid library production kit (Epicentre Biotechnologies) according to the manufacturer's protocol.

PCR screening of metagenomic DNA

In general, three primer pairs for type I PKSs were used: KSDPQQF (5'-MGN GAR GCN NWN SMN ATG GAY CCN CAR CAN MG-3') and KSHGTGR (5'-GGR TCN CCN ARN SWN GTN CCN GTN CCR TG-3') for the KS domains (Piel, 2002), AT1F (5'-TTY CCN GGN CAR GGN NSS CAG TGG-3', binding to the motif FPGQGsQW) and AT3R2 (5'-GC IGC IGC NAT CTC NCC C-3', binding to the motif QGEIAAA) for the AT domains, and SWF_ATF (5'-TTC TCC GGG CAG GGC ACG CAG TG-3', binding to the motif FSGQGTQW) and SWF_ATR (5'-CAG TTC CAC CAG CGC GCA CTG-3', binding to the motif QCALVEL) designed to be specific for the AT domain of *swfA*. To obtain PCR products, 0.5 µL of sheared (by pipetting 100 times up and down) metagenomic DNA was used in a 50 µL reaction [27 µL H_2O, 2 µL $MgCl_2$ (25 mM), 3 µL DMSO, 1.5 µL dNTP (10 mM), 5 µL primers (10 mM), 5 µL Taq buffer advanced (Eppendorf), 1 µL RBC Taq DNA polymerase (5 U/µL, RBC Bioscience)]. The cycler program was 1) 94 °C for 45 s, 2) 94 °C for 1 min, 3) 54 °C/ 56 °C/ 58 °C for 1 min in case of KS primers, and 58 °C/ 60 °C/ 62 °C for 1 min in case of AT primers, respectively, 4) 72 °C for 45 s, repetition of 2), 3), and 4) 30 times, 5) 72 °C for 7 min, 6) 4 °C forever. The PCR products were purified from the agarose gel using QIAquick gel ex kit (Qiagen), subcloned via T/A cloning into pBluescriptII SK(+) (Stratagene), and transferred to electrocompetent cells. After blue white screening plasmid preps of white colonies were digested with *Hae*III and *Msp*I for RFLP analyses. Inserts of representative plasmids were sent to GATC Biotech AG (Konstanz,Germany) for single read sequencing using the T7 primer.

Fosmid sequencing

The fosmid pPS11G3 was found with AT1F/AT3R2 primers and shotgun sequenced. A new fosmid pPS2D9, was isolated from the library with 11G3_SAM_up (5'-ACC CGA AGC AGC CTC CCA CCT ACT-3') and 11G3_SAM_low (5'- CCC CGC GAG AAC TGC AGA CAC ATC-3') primers and was shown to contain the complete cluster by end sequencing of the insert. To close the gap of 11G3, 11G3_close_gap_up (5'-CGA AGA CCG CTC CTT CCT C-3') binding to EDRSFL and 11G3_close_gap_low (5'-GCC GCT GAC CGG CAC TCT-3') binding to PLTGT were used on the template of pPS2D9. For primer walking to obtain the end of 11G3 the primer 2D9_restSAMup (5'-AGC AGA TGG TGG CCG AGT TCG ACT-3') was used. The second completely sequenced fosmid (pPSA11D7) was isolated from the library using ATPEF (5'-ATG GTG TTT TCG GGG CAG GGC ACG CA-3') MVFSGQGTQ and ATPER (5'-GGC GGC GGC CAC CTC GCC CGA GCT GTG TCC-3') GHSSGEVAAA. More representatives were isolated from the library using A11D7_54F (5'-GTT TCC TGG GAC ACC TTC AG-3') and A11D7_530R (5'-GGT GAG CTT TGC GTT GTT G-3'). To obtain the missing final part of the sequence PSA11D7, 3i10E_lastF (5'-CGA GCC TCG CGA GTT CAC C-3') was used.

Bioinformatics

Sequences were analyzed using BLASTp and BLASTx (Altschul *et al.*, 1997) and aligned with BioEdit (Hall, 1999). Phylogenetic analyses (Neighbour Joining, 111 replicates, 1,000 bootstraps) were performed using ClustalX (Larkin *et al.*, 2007) or the MEGA 5.05 software package(Tamura *et al.*, 2011).

Cloning of the swfA$_{ACP}$ domain

The sequence of the ACP domain of *swfA* was amplified by PCR using primers 11G3_ACP_up (5'-AAA GGA TCC ctg acg ctc gaa ggt gtg gt-3') and 11G3_ACP_low (5'-AAA AAG CTT **TCA** gtc ggc ccc gtg cac gat tgc c-3'). (Introduced restriction sites are underlined and Stop codon is in bold.) The 234 bp long PCR product was subcloned via T/A-cloning in pBluescript II SK(+) (Stratagene) to yield pGS21, and cut out from the vector with BamHI and HindIII. The fragment was then ligated into two different vectors: (i) a pHIS8 vector with an additional gene for the PPTase Svp inserted into its NotI site under the control of the same promoter as the ACP (i.e., pHIS8-Svp, to yield pGS30), and (ii) a pHIS8 vector without this gene (i.e., pHIS8, to yield pGS34). The history of pHIS8-Svp has been described in the literature (Jez *et al.*, 2000; Izumikawa *et al.*, 2006).

Protein expression and purification

Proteins from plasmids pGS30 and pGS34 were expressed as N-terminal His8-tag fusion proteins and purified over Ni-NTA columns. To do so, *E. coli* BL21-CodonPlus®(DE3)-RIPL electrocompetent cells (Stratagene) were transformed with the expression plasmid and spread out on LB agar plates containing 50 µg/mL kanamycin (Kan50) and grown o/n at 37 °C. From this plate, 4 clones were picked to prepare o/n cultures in LB + Kan50. 5 mL of this o/n cultures were used to inoculate 200 mL LB medium without antibiotics. The cultures were incubated at 37 °C for 2-3 h shaking with 250 rpm (OD_{600} ~0.6-0.8). After cooling to 16 °C, the cultures were induced with 1 mM IPTG and incubated for further 24 h at 16 °C. After harvesting by centrifugation, the cell pellets were dissolved in 3 mL lysis buffer (50 mM Tris-Cl, 500 mM NaCl, 10 mM $MgCl_2$, pH 8.0) and frozen o/n. After thawing on ice and ultrasonication, the lysates were obtained by centrifugation for 20 min at 15,000 rpm in a table top centrifuge at 4 °C.

The supernatants were transferred to 15 mL falcon tubes and after addition of 300-600 µL PerfectPro® Ni-NTA agarose (5PRIME) incubated on ice for 1 h under horizontal shaking; the pellets were (partly) resuspended in 1 mL of lysis buffer by vortexing. To obtain purified soluble proteins, the lysate/Ni-NTA Agarose mixtures were transferred to Poly-Prep® chromatography columns (Bio-Rad) that fit to a SPE chamber (Supelco). Elution fractions (500 µL each) were obtained by stepwise increasing the imidazole concentration in the lysis buffer from 0 (= wash) to 300 mM. Aliquots of 50 µL were mixed with 25 µL of 3× SDS loading buffer [2.4 mL Tris-Cl (1 M, pH 6.8), 3 mL SDS (20%), 3 mL glycerol, 1.6 mL β-mercaptoethanol, 6 mg bromophenol blue, and 10 mL H_2O], incubated for 5 min at 99 °C, spun down shortly and ~20 µL of elution fractions were loaded on mini SDS-PAGE (Figure S4).

The purified proteins were desalted 3 times with milli-Q®-H_2O in VivaSpin500 centrifugation units (Sartorius, 5000 MWCO), diluted 1:2 with MeOH, containing 0.2% formic acid, and subjected to FTMS analysis.

Cloning and in vitro expression of the swf cluster in E. coli

A region upstream the POR_0546 homolog (22091-22720,~1000 bp upstream *supE* permease) was amplified by PCR from the fosmid pPS2D9 using primers: 5'-AAA GGA TCC tga cca cgc cct cgt gct ca-3' (forward) and 5'-AAA GAA TTC tgg acc act tcc ggc aac tac-3' (reverse), ligated into pBluescript II SK(+) via T/A cloning to yield pGS15, and cut out from the vector with *Bam*HI and *Eco*RI. Another region at the end of the *swf* cluster (i.e. *swfC* 36006-37813) was amplified from pPS2D9 with the primers 5'- AAA GAA TTC tcg aac gcc ctc ctc atc tac c-3' (forward) and 5'- AAA AAG CTT gcc cac tac gtg ctg cat cgg-3' (reverse), ligated into pBluescript II SK(+) via T/A cloning to yield pGS29, and cut out from the vector with *Eco*RI and *Hin*dIII. Both inserts were ligated in a three-point ligation into the *Bam*HI/*Hin*dIII sites of pHIS8-Svp. The resulting plasmid (pGS27) was linearized with *Eco*RI and dephosphorylated. Homologous recombination was performed with this linear fragment (Kan^R) and the fosmid pPS2D9 (Cam^R) as a donor in the host strain *E. coli* BW25113 (Datsenko and Wanner, 2000) with the *red* helper plasmid pKD46 (Amp^R). Transformants were selected on LB + Kan50 agar plates and contained the whole *swf* cluster in the vector pHIS8-Svp (i.e. the plasmid pGS38). The plasmid was introduced by electroporation into *E. coli* BAP1 (Pfeifer *et al.*, 2001), which is a derivative of *E. coli* BL21-(DE3) including a gene for the 4'-phosphopantheteinyltransferase Sfp (Lambalot *et al.*, 1996).

The strains were grown in baffled 500 mL Erlenmeyer flasks in 100 mL MMGAGTr medium supplemented with 50 µg mL^{-1} kanamycin. MMGAGTr was prepared as follows: 200 mL M9 salts (64 g Na_2HPO_4 x 7 H_2O, 15 g KH_2PO_4, 2.5 g NaCl, 5.0 g NH_4Cl, *ad* 1000 mL milliQ H_2O), 500 µL of 2000X trace element solution (1.2 g $FeCl_3$ x 6 H_2O, 1.4 g $MnSO_4$, 1.6 g $CuSO_4$, and 500 mL milliQ H_2O), 2 mL 1 M $MgSO_4$, 100 µL 1 M $CaCl_2$, 10 g glucose, 5 g L-glutamic acid, *ad* 1000 mL milliQ H_2O. Four clones of transformants were picked from the LB plates to inoculate 2.5 mL MMGAGTr medium in one test tube for overnight cultures (37 °C, 250 rpm) in order to inoculate the 100 mL medium in EM-flasks next day. After 2-3 h of shaking (250 rpm) at 37 °C the *T7* promoter was induced with 0.1 mM IPTG, and 5-10 nM cobalamin (vitamin B12) was added as a co-factor of SwfC. The cells and the culture broths were harvested separately after 3 h of further growth at 30 °C, 250 rpm. The cell pellet was dissolved in 1 mL of nanopure water, frozen o/n, and after thawing on ice and ultrasonication, the lysates were subjected to extraction with 4 mL of MeOH. Culture broths were freeze dried o/n and extracted with 5 mL of MeOH/H_2O (8:2). All the experiments were performed in triplicates.

LC-HR-ESI-MS analyses

The experiments were performed using a Thermo LTQ Orbitrap XL high-resolution ESI mass spectrometer coupled to an Agilent model 1100 LC, which included a solvent reservoir, in-line degasser, binary pump, and refrigerated autosampler. A 2.6 μm Kinetex C18 column (50 × 2.1 mm), maintained at room temperature, was used. It was eluted at 150 μL min^{-1} with H_2O and CH_3CN, using as a gradient elution 70–95% CH_3CN over 23 min and hold 13 min. Crude extracts from transformants and their culture broths were filtered and injected (5 μL) without any further workup. Both positive-ion and negative-ion mass spectra were recorded in separate HPLC runs.

References

Altschul, S.F., Madden, T.L., Schaffer, A.A., Zhang, J., Zhang, Z., Miller, W., Lipman D.J. (1997) Gapped BLAST and PSI-BLAST: a new generation of protein database search programs. *Nucl Acids Res* **25:** 3389-3402.

Datsenko, K.A., Wanner, B.L. (2000) One-step inactivation of chromosomal genes in Escherichia coli K-12 using PCR products. *Proc Natl Acad Sci U S A* **97:** 6640-6645.

Hall, T. A. (1999) BioEdit: a user-friendly biological sequence alignment editor and analysis program for Windows 95/98/NT. *Nucleic Acids Symp Ser* **41:** 95-98.

Izumikawa, M., Cheng, Q., Moore, B.S. (2006) Priming Type II Polyketide Synthases via a Type II Nonribosomal Peptide Synthetase Mechanism. *J Am Chem Soc* **128:** 1428-1429.

Jez, J.M., Ferrer J.L., Bowman M.E., Dixon R.A., Noel J.P. (2000) Dissection of malonyl-coenzyme A decarboxylation from polyketide formation in the reaction mechanism of a plant polyketide synthase. *Biochemistry* **39:** 890-902.

Lambalot, R.H., Gehring, A.M., Flugel, R.S., Zuber, P., LaCelle, M., Marahiel, M.A. *et al.* (1996) A new enzyme superfamily. The phosphopantetheinyl transferases. *Chem Biol* **3:** 923-936.

Larkin, M.A., Blackshields, G., Brown, N.P., Chenna, R., McGettigan, P.A., McWilliam, H. *et al.* (2007) Clustal W and Clustal X version 2.0. *Bioinformatics* **23:** 2947-2948.

Pfeifer, B.A., Admiraal, S. J., Gramajo, H., Cane, D. E., Khosla, C. (2001) Biosynthesis of complex polyketides in a metabolically engineered strain of E. coli. *Science* **291:** 1790-1792.

Tamura, K., Peterson, D., Peterson, N., Stecher, G., Nei, M., Kumar, S. (2011) *Mol Biol Evol* **28:** 2731-2739.

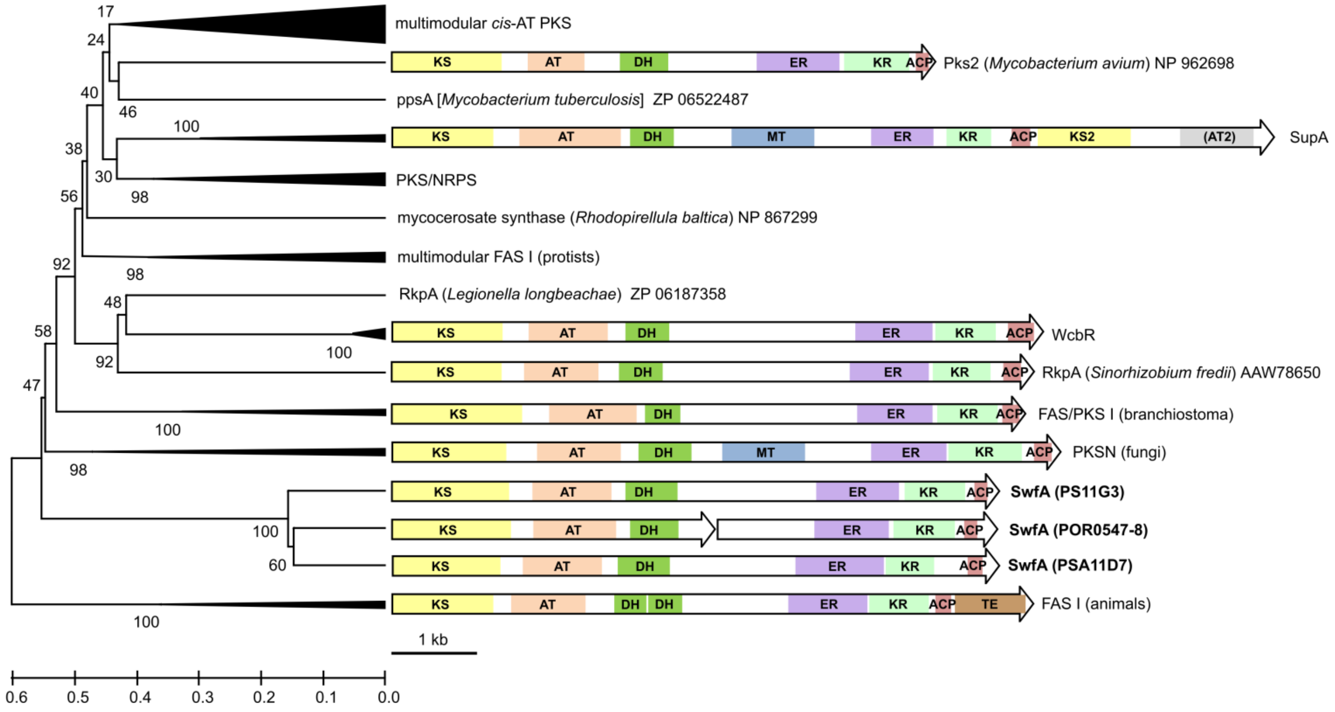

Figure S1. UPGMA tree showing phylogenetic relationships of KS-AT domains from different types of PKS and FAS enzymes, and in-scale sketches of domain architectures. Branches comprising multiple sequences have been collapsed and are illustrated as triangles; bootstrap values are shown at the nodes.

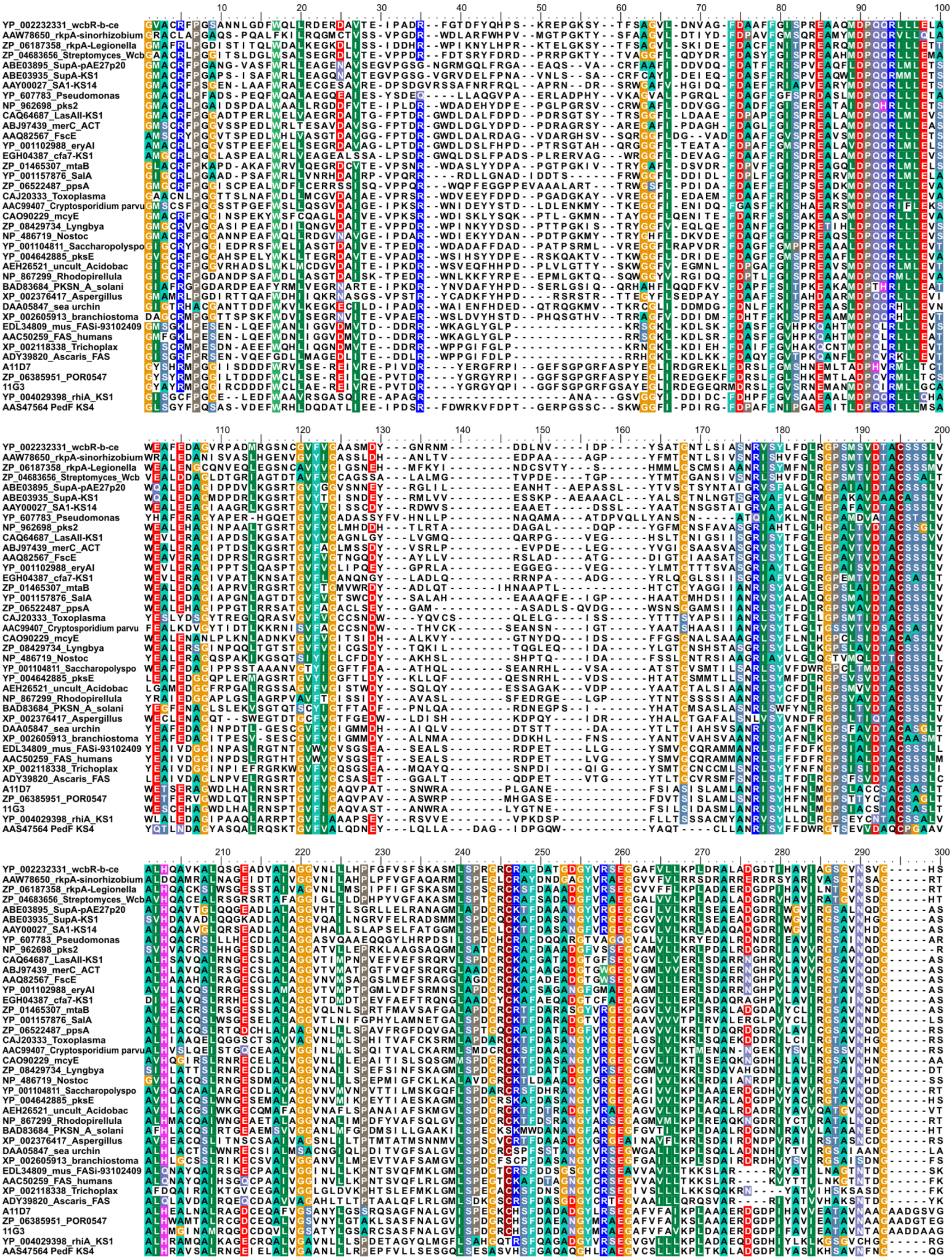

Figure S2. Alignment of KS domains from type I PKSs and FASs. The KS domain of SwfA shows features that are not shared by any other KS domains, including WcbR/RkpA. These are, for instance, the triple "D" at position 8-10 aa, the "EATAVN" motif (aa 250-255) and 8 additional aminoacids after aa 275, and the NGHCVVR motif at the end (aa 413-419). In addition, the second "Q" in the DPQQR motif is either "I" or "V", which is similar to animal FAS I and not to PKSs (all *cis*-AT and WcbR/RkpA sequences possess an intact DPQQR motif), and the motif HGTGT of *cis*-AT and WcbR/RkpA sequences is changed to H**A**TGT, with the A being unique among all KS sequences.

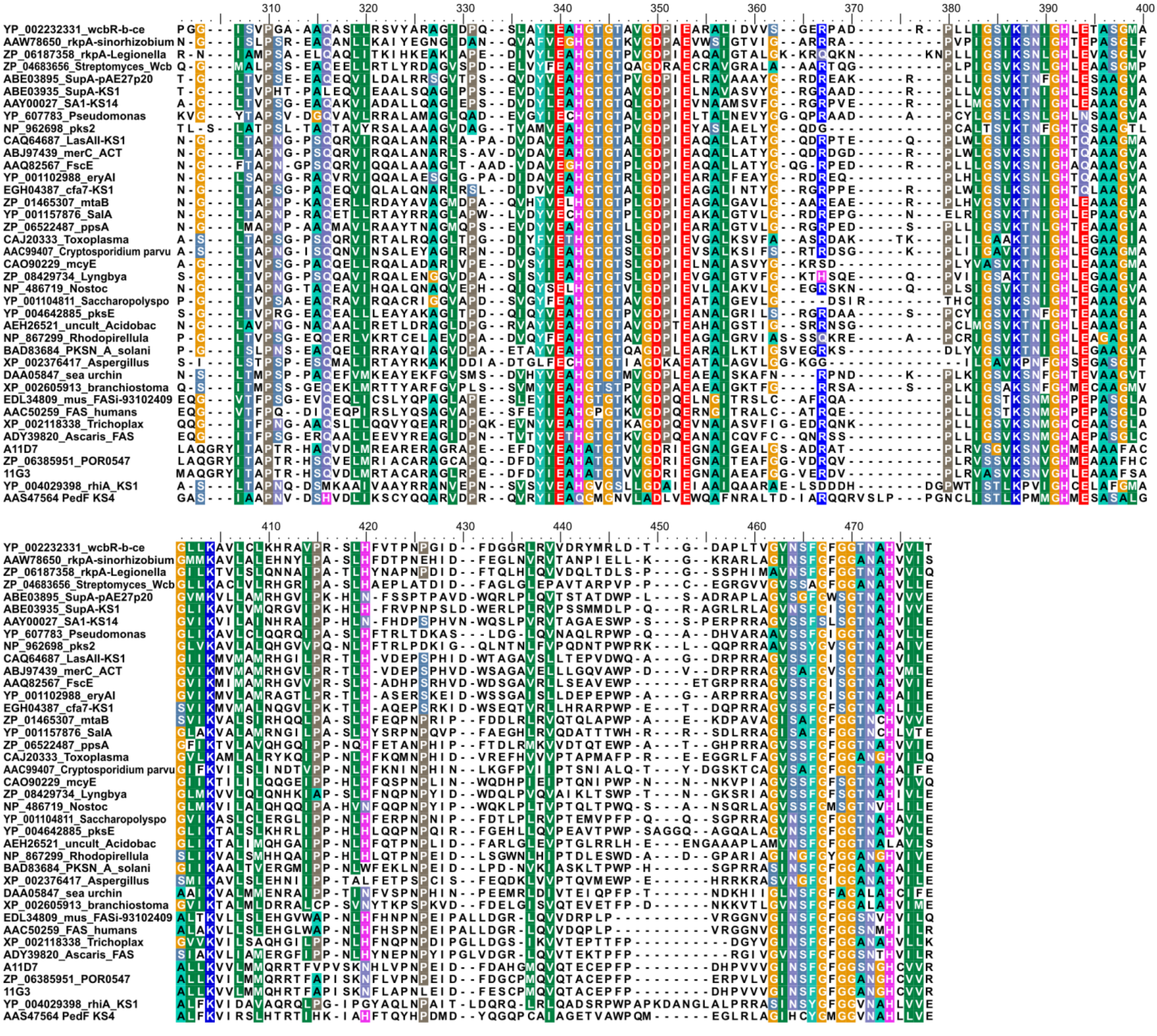

Figure S2 (continued)

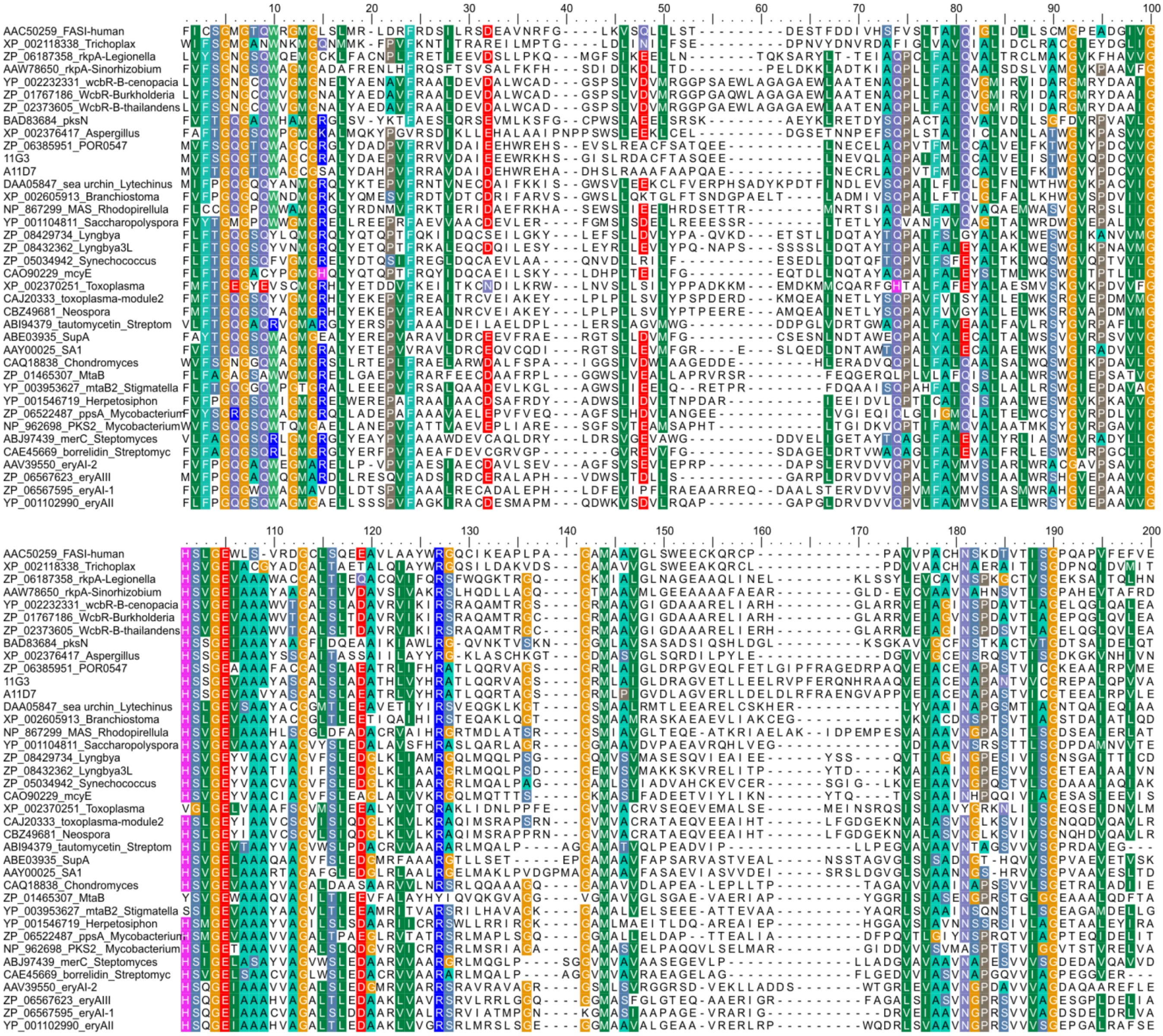

Figure S3. Alignment of AT domains from type I PKSs and FASs.

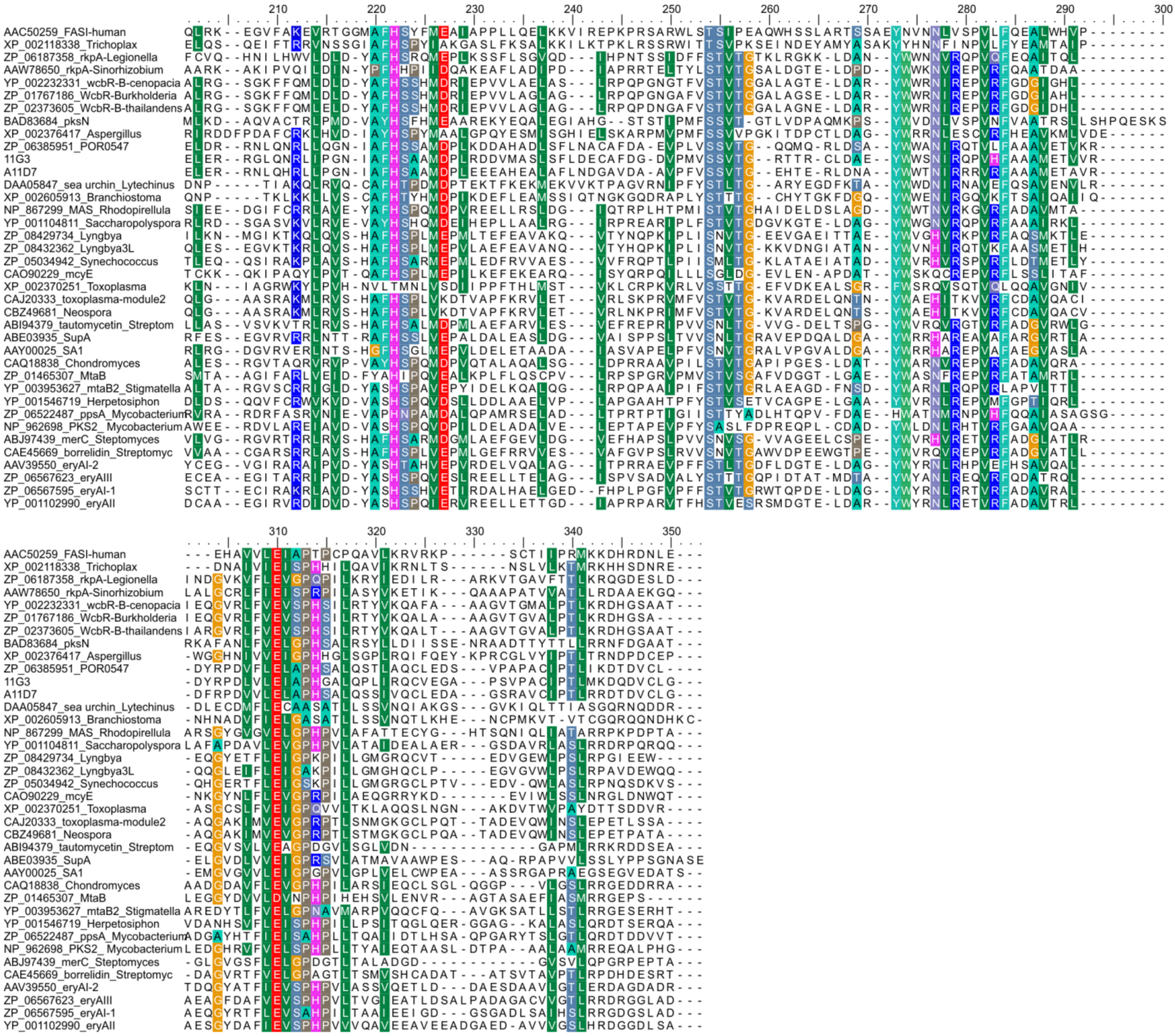

Figure S3 (continued)

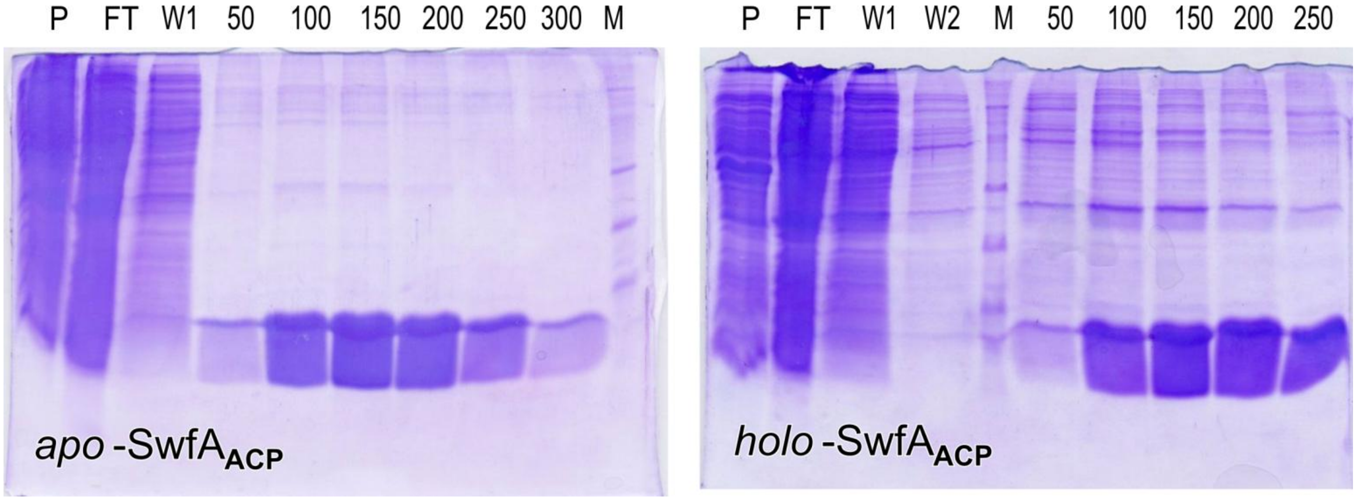

Figure S4. The proteins *apo*-SwfA$_{ACP}$ and *holo*-SwfA$_{ACP}$ were expressed as N-terminal His8-tag fusion proteins and purified over Ni-NTA columns. Elution fractions (500 µL each) were obtained by increasing the imidazole concentration in the lysis buffer from 0 to 300 mM stepwise. SDS-PAGE gel was loaded with resuspended cell pellet (5 µL, lane P), column flow through, containing unbound proteins (12 µL, lane FT), washing fractions eluted with lysis buffer (20 µL each, lanes W1 and W2), and fractions eluted with increasing (50–300 mM) imidazole concentrations (20 µL each, lanes 50,100,150, 200, 250, and 300). All the fractions were previously mixed with 3X SDS loading buffer. Lane M is a pre-stained protein marker (Roti®-Mark STANDARD).

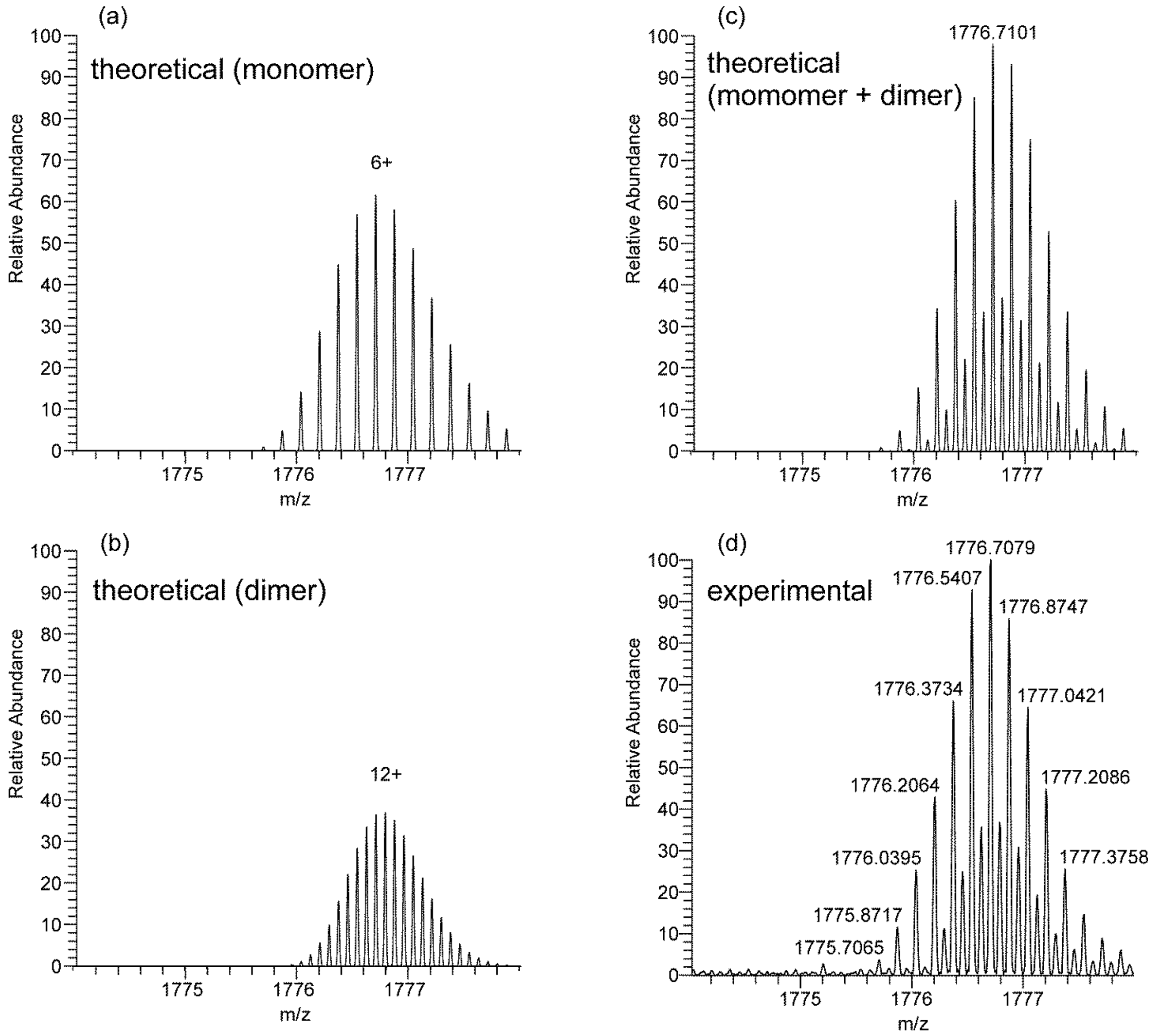

Figure S5. Analysis of the fine structure of the pseudomolecular ion peak of *holo*-SwfA$_{ACP}$ at *m*/*z* 1776.7, showing the simultaneous presence of the a 6+ ion of the monomer and a 12+ ion of the dimer. (a) Theoretical spectrum of the 6+ ion of *holo*-SwfA$_{ACP}$ radical, molecular formula $C_{460}H_{730}N_{136}O_{146}PS_4^{6+}$, calculated using the program IsoPro 3.0; (b) Theoretical spectrum of the 12+ ion of *holo*-SwfA$_{ACP}$ dimer, molecular formula $C_{920}H_{1460}N_{272}O_{292}P2S_8^{12+}$. The ions of the monomer and of the dimer have the same average *m*/*z*, but a different charge and a different isotope pattern; (c) Sum of the theoretical spectra of the monomer and dimer. (d) Experimental spectrum.

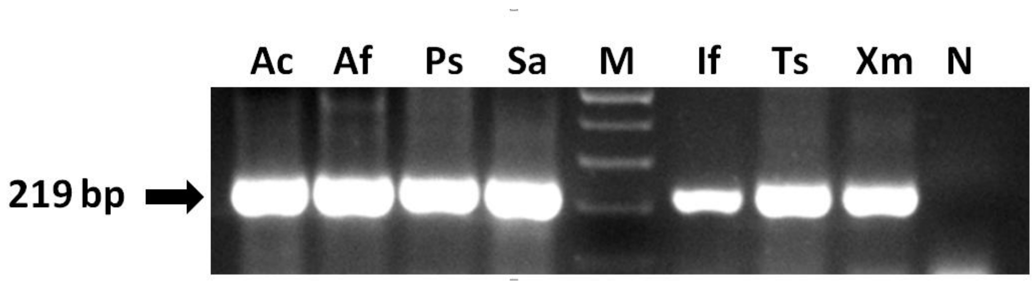

Figure S6. PCR detection of *swf* genes in seven different "high microbial abundance" marine sponges. Primers SWF_ATF and SWF_ATR designed on SwfA$_{AT}$ were used. Abbreviations: Ac, *Aiolochroia crassa*; Af, *Aplysina fulva*; Ps, *Plakortis simplex;* Sa, *Smenospongia aurea*; M, DNA size marker; If, *Ircinia felix*; Ts, *Theonella swinhoei*; Xm, *Xestospongia muta*; N, negative control.
